# Compensation Techniques Aimed at Mitigating Vibrations in Optical Ground-Based Telescopes: A Systematic Review

**DOI:** 10.3390/s21113613

**Published:** 2021-05-22

**Authors:** Guillermo Palacios-Navarro, Fernando Arranz Martínez, Raúl Martín Ferrer, Pedro Ramos Lorente

**Affiliations:** Department of Electronic Engineering and Communications, University of Zaragoza, 44003 Teruel, Spain; farranz@unizar.es (F.A.M.); ramar@unizar.es (R.M.F.); pramos@unizar.es (P.R.L.)

**Keywords:** adaptative optics, closed-loop control, ground-based telescopes, optical telescope, vibration mitigation, vibration attenuation

## Abstract

The objective of this study was to evaluate the performance of the different systems and techniques aimed at suppressing vibrations on optical ground-based telescopes. We identified the studies by searching three electronic databases (Science Direct, IEEE library and Web of Science) from the year 2000 to December 2020. The studies were eligible if they proposed systems focused on mitigating the effects of vibrations in optical telescopes and brought performance data. A total of nine studies met our eligibility criteria. Current evidence confirms the feasibility of adaptative optics (AO) systems based on closed-loop control to mitigate vibrations, although variations and additions should be made depending on their nature and characteristics in order to improve the performance of the proposed techniques. This systematic review was conducted to provide a state-of-the-art of the methods and techniques that have been developed over the past two decades. The review also points out some issues that demand future research.

## 1. Introduction

The quality of the images captured in professional astronomy is of vital importance to the scientific community, so a high degree of accuracy is required in the technology used to take them [1,2,3,4,5]. This technology, in addition to providing images of the highest possible quality through their respective charge-coupled devices (CCDs), must be able to mitigate all sources of noise that cause a loss of image quality. There are different natural phenomena that influence and reduce the performance of a certain telescope. We have divided them into three groups: atmospheric turbulence, natural phenomena and vibration induced by the telescope’s mechanical components.

### 1.1. Atmospheric Turbulence

Although it is not a source of vibrations by itself, the atmosphere introduces aberrations in the wavefronts. A more turbulent and unstable atmosphere will introduce stronger aberrations in the wavefronts, which will result in a poor imaging quality compared to a more relaxed atmosphere. This phenomenon is known as atmospheric optical turbulence and has its origin in the random variations of the refractive index associated with temperature [6]. These temperature homogeneities are governed by the Kolmogorov–Obukhob turbulence law [7,8]. The phenomenon has a high degree of affection in astronomical observations, since the wavefront is no longer flat when it reaches the observer on the ground, therefore limiting the capabilities of ground-based telescopes [9,10,11].

### 1.2. Natural Phenomena

On the other hand, wind loading is also an important element that can cause vibrations in the telescope. Telescope wind shake was estimated to approximately 300 milliarcseconds (mas) rms in the European Extreme Large Telescope (E-ELT) [12]. In fact, the wind-induced pounding and the turbulences it can generate by itself in the telescope dome are usually taken into account in the design of these structures [13]. The response due to this wind load depends critically on the lowest structural eigenmode [14]. A characterization of the vertical wind speed distribution represents a parameter to take into account for an astronomical site when designing an AO system [15]. Tichkule and Muschinski studied the effects of wind-driven telescope vibrations on optical turbulence measurements. In particular, on optical angle-of-arrival (AOA) fluctuations [16].

It is also interesting to note that given the construction characteristics of large telescopes, in addition to the resonance frequency that the telescope pier (together with the telescope itself) may have, it can be influenced by the dynamic interaction between the soil and the telescope pier, causing changes in the first resonance frequency of the telescope pier [17]. François et al. [14] performed an analysis of this interaction of the soil with the telescope structure at the Javalambre Astronomical Observatory (JAO) and in their analysis found that the first eigenfrequency of the telescope pier shifted down from 14.3 Hz to 11.2 Hz, thus demonstrating a significant effect on the soil–structure interaction.

Other sources of vibration are found in the ground (ground vibrations). For example, seismic activity caused by tectonic plate movement or volcanic activity could induce subtle ground tilt and this could result in misalignment of the mechanical and optical components [18]. Telescopes must withstand these effects and also minimize operating times. In addition, gases and ash or dust associated with volcanic activity can affect telescope systems in many ways: corrosion of mirrors, damage of control systems, the computer, data transmission devices, or they can affect telescope mountings, among others.

### 1.3. Vibration Induced by Mechanical Elements

In addition to all the above, there are disturbances due to other elements, such as those caused by shutters, cooler fans, cooling systems and motors, telescope orientation, telescope tracking errors, actuator imperfections, among other sources. They can be classified as structural vibrations. After atmospheric turbulence, telescope vibrations are the strongest disturbance that telescope instrumentation has to face. In spite of the fact that they usually are low-frequency vibrations (due to the mass of telescopes), their amplitudes present a real stumbling block when it comes to correcting them [19]. These vibrations due to the telescope’s own structure and operation can be very important. For example, the fact that the structures supporting the mirrors are light compared to the weight of the telescope itself, causes resonances to occur in these structures between 10 and 100 Hz [20]. Another example is the Gemini Planet Imager [21], at the Gemini South telescope, which is an instrument with an Extreme Adaptive Optics (XAO) system dedicated to the detection of exoplanets, and very sensitive to vibrations. After four years of characterizing a vibration present at around 60 Hz, it was determined that the most significant source was from the instrument’s cryogenic coolers [22].

When we face the problem of vibration attenuation, we find different types of spectra. There are two main types: either a spectrum with few very sharp and properly separated structural modes, or a spectrum with many different modes and damped to different extents, giving rise to a very wide frequency spectrum with smeared peaks [20].

Lozi et al. [19] studied the vibrations in the Subaru telescope by means of accelerometers, using the Subaru Coronagraphic Extreme Adaptive Optics (SCExAO) instrument, and found that the vibrations were introduced by the telescope itself and not the instrumentation. Specifically, they were related to the encoders of the telescope’s drive system, both in altitude and azimuth.

The distortions caused by the above phenomena translate into a poor quality of the images obtained by the telescopes. In recent years, AO systems have been designed and implemented in most large ground-based telescopes. These systems are essential to achieve much higher angular resolutions and thereby improve the quality of the images [23]. AO systems have proven to be robust in compensating for the effect of wavefront distortion due to atmospheric turbulence by using deformable mirrors [24,25,26,27] as well as for mitigating vibrations due to the telescope structure [16,28,29]. Even so, there is a level of disturbance energy that still remains and needs to be minimized, since disturbances affect the correction of the wavefronts. The existing literature shows that much work has been done on the mitigation of disturbances in tip–tilt modes that originated from mechanical vibrations associated with the telescope structure and its instrumentation [30,31,32,33]. This is because tip–tilt aberrations are the main contributors to wavefront deformation.

The article is organized as follows. The second section is focused on the description of the methods used to fulfill this review. Section three presents the search results, the characteristics of the selected studies together with the outcome measures and a brief summary of every study included in the review. Section four presents the discussion of the obtained results together with some unresolved issues that required further research. Section five summarized the main findings.

## 2. Methods

### 2.1. Eligibility Criteria

We included studies that proposed systems aimed at mitigating the effects of vibrations in optical telescopes, regardless of their origin. Studies were accepted when they were published in a peer-reviewed journal and they were written in English. To be included in the review, the studies had to present some measures of performance. We excluded systems applied on other telescopes, such as radio telescopes or space telescopes, since our study is circumscribed to ground-based optical telescopes. Studies that only investigated the characterization of the vibrations on telescopes were also excluded.

### 2.2. Search Procedure

We searched different databases electronically: (IEEE Electronic Library, Science Direct) from the year 2000 until December 2020. The major search terms were telescopes and vibrations. Depending on the search engine, subject headings and keywords based on the search terms were used to identify relevant articles. To summarize, we attempted to obtain publications that contained different techniques and systems aimed at mitigating the effects of vibrations in optical telescopes. Reference lists from the identified publications were also reviewed to identify additional research articles of interest.

### 2.3. Data Collection

Two review authors (G.P.-N and F.A.M.) independently reviewed the titles and abstracts retrieved from the search in order to determine if they met the predefined inclusion criteria. The full text was checked in cases of uncertainty. Two review authors (P.R.L. and R.M.F.) moderated any disagreement. The full text articles were analyzed in order to extract the most relevant features.

### 2.4. Outcome Measures

The main outcomes found in the studies were the following: the Strehl Ratio (SR), the Encircled Energy (EE), the relative Long Exposure Strehl (LE Strehl), the cumulative power spectral density (PSD), tip–tilt and jitter residuals, the Optical Pathway Difference (OPD), as well as attenuation percentages related to original vibration values or error percentages with respect to the original errors.

## 3. Results

### 3.1. Search Results

**Figure 1:**
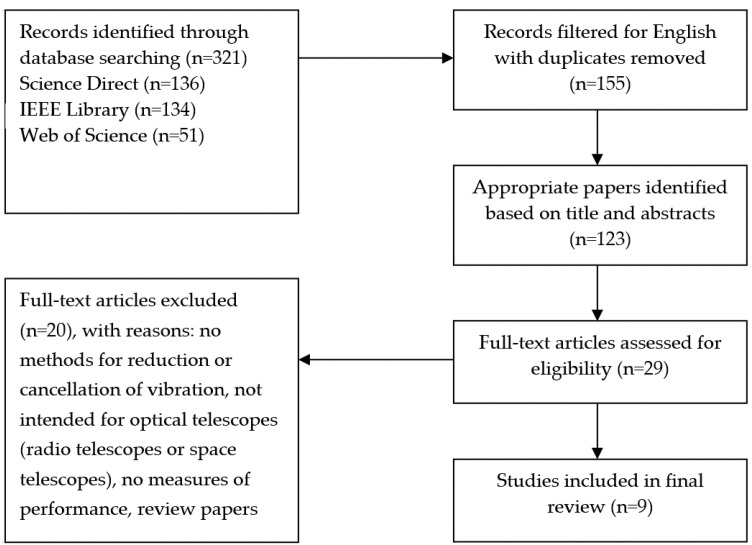
Consort diagram of study selection.

The initial search yielded 321 articles. After removing duplicates, 155 potential articles that investigate the effects of vibrations on telescopes were identified. The authors independently evaluated the titles and abstracts, taking into account the inclusion and exclusion criteria. Whenever necessary, a more thorough study was carried out in order to discard articles that did not match the established criteria. Finally, the population of our study consisted of nine articles. All of them refer to the different methods and techniques to mitigate/cancel/suppress vibrations on telescopes regardless of the origin of such vibrations. The details of the search result are summarized in Figure 1.

### 3.2. Characteristics of the Selected Studies

After selecting the articles, we extracted the following variables: concerning authors, country and year of publication, type of telescope the focus was on, vibration origin, sensing technology and sensor location, goal of study, techniques and methods used together with the range of frequencies, results and main findings. The details of the general characteristics of the different studies are summarized in Table 1. The included studies took place in three countries: three studies took place in Germany [20,34,35], four in China [32,36,37,38] and one in Italy [39]. The study of Muradore et al. [40] was an Italian–German cooperation.

(a) Type of telescope. Regarding the type of telescope for which the research is directed, four studies refer in their experiments to the Large Binocular Telescope (LBT)) [20,34,35,39], although all of them are based on laboratory simulations. Muradore et al. [40] tested their algorithm at the ESO-VLT. The systems presented by Niu et al. [37] and Tang et al. [32,36,38] refer in general to optical telescopes with AO control. 

(b) Origin of the vibration. All research is focused on trying to reduce the amount of structural vibration. In some studies, signals were also introduced to simulate the behavior of atmospheric turbulence through a model different from the models used to represent structural vibrations, such as Agapito’s [39] and Muradore’s study [40]. On the other hand, Böhm et al. [20] uses a model to simulate the telescope dynamics in which the wind loading effect is included. Finally, the wind effect is also considered in the study of Agapito [39].

(c) Sensing technology. Six of the studies [32,36,37,38,39,40] use image sensors. Of these, Agapito et al. [39] uses a pyramidal WFS, while Muradore et al. [40] uses a WFS. Three of the studies [20,34,35] use an array of accelerometers usually located on the surface of the telescope mirrors, in order to reconstruct the vibrational modes. 

(d) Study goals. Vibration reduction in the tip–tilt–mirror modes (tip–tilt–piston mirror) is the focus of most studies [32,34,36,37,38,39,40], since these modes quantify the image displacements in two orthogonal directions [39]. Böhm’s studies [20,35] were focused on the compensation of piston aberrations by minimizing the OPD. 

(e) Frequency range operation. In general, the range of vibration frequencies to be suppressed does not go beyond 60 Hz. Since most of the studies focus on suppressing frequencies of tip–tilt modes, the range is narrower, specifically the vibration frequencies are concentrated in the range 0–21 Hz. The frequency spectrum in the study of Niu et al. [37] ranges from 7.5 to 12.5 Hz; Tang’s et al. study [38] ranges from 0 to 11 Hz; whereas the studies by Tang et al. [36] and Agapito et al. [39] range from 0 to 20 Hz. In the case of studies on larger telescopes, such as LBT or VLST, the frequency range spans further. In Böhm’s study [20] the frequency range goes up to 80 Hz, although the OPD was reduced about 80% in the range 14–20 Hz. In Muradore’s study [40], the study range reaches 200 Hz, although the most important vibration peaks occur at frequencies of 18 and 48 Hz, respectively. In the study of Tang et al. [32], despite having to peak at frequencies of 22 Hz and 78 Hz, the energetic bandwidth lies below 10 Hz, with two peaks at 4.8 and 6 Hz.

(f) Vibration suppression methods and experimental setups. All studies employ loopback techniques for the control of AO systems. The improvements introduced in the different control systems are compared with the results that would be obtained with classical integral (or classical proportional integral) control. Some studies use mixed-control approaches, combining classical and observer-based techniques, such as that of Agapito [39], in which the tip–tilt modes are controlled with a DOB (based on Kalman or H∞ filters) and the remaining modes are controlled with a simple integrator-based controller. There are studies that employ the state-space model formalism, such as observers based on linear quadratic gaussian (LQG) control laws with the use of Kalman or H∞ filters as estimators, such as those of Agapito [39] and Glück [34]. Three studies [36,37,38] use DOB together with add-on controllers based on low-pass Q filters and notch filters to cancel vibration peaks. Tang et al [32] introduced a Youla–Kucera-based controller added in a classical loopback control. In some studies [20,34,35], accelerometer-based disturbance feedforward (DFF) subsystems are also added. The two studies of Böhm [20,35] were focused on the LBT interferometer; this is why they focused their study on the reduction of the differential piston between the two telescopes that provided the beam to the interferometer (the piston does not affect single-telescope imaging). The study of Glück [34] also compares the performance of the FFD system with classical integral control with the LQG technique. Muradore et al. [40] use a fully recursive adaptive algorithm to reject vibration in the AO system. 

Regarding the experimental setup configuration, we also found a common pattern. Thus, for the application of the different vibration level reduction techniques, all the studies develop an experimental setup in the laboratory, obtaining results based on smaller optics and actuators of similar characteristics and even with small telescopes, in order to reflect the effects that can be produced on a large scale. It must be taken into account that the actuators that intervene directly or indirectly in the optics and instrumentation of large telescopes must have very specific control specifications in order not to exceed the mechanical limits of acceleration, which could damage their optics or sensitive instrumentation. That is why the algorithms and tests are performed in laboratory setups, and once verified and validated, they are debugged on the large telescopes in tests with real equipment. However, of all the studies analyzed in this review, only one [39], in addition to performing laboratory experiments, performs an implementation somewhat closer to reality. This is the case of the study of Agapito et al. [39], who, in addition to the verification of results in the experimental laboratory setup, tested the developed system in real working conditions in the solar tower of the Arcetri Astrophysical Observatory. For this experiment, the LBT optical bench (including the ASM and the WFS) had to be installed between the pillars of the tower and in the room.

### 3.3. Outcomes Measures

Regarding the results offered by the different studies as performance criteria, we highlight the following: the SR factor, which appears as a measure of performance in the system of Agapito [39] and Glück [34]; the latter also calculates the Encircled Energy (EE) for the different control systems and bright NGS and faint NGS, respectively. The relative Long Exposure Strehl (LE Strehl) is quantified in the study by Muradore [40]. The cumulative PSD function is also used to demonstrate the goodness of Muradore’s system [40]. In the latter study, the tip–tilt and jitter residuals, respectively, are also quantified. In Böhm’s study [20], the OPD is obtained because it is intended for the LBT interferometer. Other studies present the results as attenuation percentages with respect to the original vibration values [20,35,37,39]. Finally, in two studies [32,38] the percentage with respect to the original error is reflected. Below, we briefly describe the studies included in the analysis together with their main findings.

Niu et al. [37] designed and implemented a new technique for structural vibration mitigation in the tip–tilt mirror system based on an EDOB system (based on a Youla parameterization), together with an improved Q filter to which they add several notch filters. The reason for using this parameterization is to improve the vibration rejection capability of the conventional control structures. To this end, it uses an image sensor to provide the position error for the control of a piezoelectric sensor acting on the tip–tilt mirror system. The experimental results show progressive improvements as more notch filters are added, reaching a 36% improvement in vibration attenuation in the tip–tilt mirror control system with the addition of three notch filters, in the considered bandwidth (7.5–12.5 Hz) and with respect to the performance offered by the conventional control loop. Thanks to its simple structure and design process, the system can be applied to other servo control systems. We also highlight its cost-effectiveness, since it uses only one image sensor.

Tang et al. [32] proposed a closed-loop control system based on a Youla–Kucera parameterization in order to optimize the vibrations in tip–tilt modes. They focused the study on the control of stability and error attenuation. In addition to this controller, the authors optimize both low-pass and high-pass Q filters to attenuate low-frequency and high-frequency vibrations, respectively. The best low-frequency attenuation results were achieved with a Q_31_ filter [41], whereas high-frequency vibrations had to be attenuated by means of two notch filters, in order to attenuate important peaks at 22 Hz and 78 Hz. They also developed a scanning method to ensure the detection of the vibrations of interest (frequency peaks), because a suitable design of the notch filters involves a good knowledge of the frequencies of the vibrations. The results can be extrapolated to other controls for deformable mirrors.

Tang et al. [36] implemented a DOB control with a single image sensor to reject structural vibrations that affect the tip–tilt mirror modes and hence the image quality. The image sensor provided the position errors for tip–tilt mirror control. The DOB control is added to the original loop in the tip–tilt mirror control system, to which an improved Q filter is also added to reject higher amplitude vibrations while attenuating amplifications derived from the controller for frequencies other than the structural vibration frequencies. In addition, its low-pass characteristic blocks unmodeled dynamics and high-frequency noise that affects closed-loop stability. The model was tested in an experimental setup, showing a vibration bandwidth between 10 and 20 Hz, with significant peaks at frequencies around 13 Hz, 17 Hz and 21 Hz. These vibration peaks are mitigated with notch filters. The results obtained show a 46.1% decrease in vibrations in tip–tilt modes with respect to the integral control loop.

Tang et al. [38] proposed the introduction of an add-on controller in the control loop of the tip–tilt mirror system to mitigate vibrations in the telescope. The proposed method uses only the tip–tilt errors from an image sensor to implement a disturbance observer, without the constraint of having a very accurate model. The performance of the system relies on the proper design of a Q filter, in order to suppress all frequency vibrations. The developed model was validated in an experimental environment and compared with the behavior of a classical PI control. The results showed a reduction in the original error by 10%, and after the introduction of three cascaded notch filters the reduction was 43% (all compared to the classical PI control).

Agapito et al. [39] focused on the study of mixed control approaches, combining classical control techniques with observer-based techniques for the control of the LBT-AO system. In particular, they estimated the time evolution of the phase perturbations due to atmospheric turbulence and vibrations acting on the tip–tilt modes. Numerical simulations showed that the controllers based on Kalman filters and H∞ achieve an SR of about 80%, much higher than those achieved by the integrating controller (30.9% SR) in the presence of atmospheric disturbance effects and structure vibrations. In the same way, both controllers were much more robust than the integral controller when there were errors in the vibration frequency of the model. To experimentally verify these simulated results, only one experimental test was performed on a Solar Tower with the mixed-Kalman. The experimental results were calculated for the SR percentage at a wavelength of 2.2 um and amplitudes greater than 20 mas, showing a much better behavior of the controller with a Kalman filter compared to the integral controller. In the first case, values close to 70% of the SR were obtained, while in the second case only 37% of the SR was reached.

Muradore et al. [40] adapted a technique for periodic disturbance rejection (first developed by Pigg and Bodson [42,43]) to reject vibrations in the AO loops. The controller design is within the family of adaptive vibration controllers (AVC). The proposed AVC algorithm consists of an add-on controller in the AO control loop. Moreover, it does not require the intervention of an operator since no knowledge of the AO system dynamics is necessary, as it is estimated by the algorithm itself. The study focused on vibration rejection in the tip–tilt mode of the mirror as well as in the focus, trefoil-x and trefoil-y modes. Different scenarios were simulated where the VCA was fed by NGS or LGS with simulated and real-time series vibrations. The reduction in the PSD function and the standard deviation of the residual were used as measures for performance evaluation. For example, in simulated environments, a rejection of the vibration peaks at 18 Hz and 48 Hz above 20 dBs (on average) in the PSD function was obtained. The results obtained were validated in real operating conditions at the ESO observatory in Paranal (Chile). In this validation, the cumulative PSD (related to the SR factor) was used as a measure to demonstrate the improvements in the tip, focus, trefoil-x and trefoil-y modes.

Böhm et al. [20] presented different solutions to reduce the effects of vibrations on the differential piston (OPD) for the adaptive camera and the interferometer at the LBT by means of an accelerometer-based feedforward (FFD) compensation technique. Specifically, two different approaches are implemented in the control loop: one to reconstruct the position of the mirror from the accelerometer measurement (model-based reconstruction) and another using a broad-band filter. The authors aimed to estimate the mirror displacements along the optical axis and thus to calculate the OPD between both sides of the telescope. After comparing by simulation, the two strategies were implemented to mitigate the vibration within the frequency range, deducing that the model-based disturbance observer was a suitable approach only when the disturbance frequency spectra contains few isolated peaks.

The estimator with the best simulation results (broad-band filtering) was implemented in a laboratory setup described by Follert et al. [44] in order to reproduce as closely as possible the conditions of the LBT’s optical path. The results showed that the filtering-based approach was more flexible and worked very well with any disturbance in the working frequency range (8–80 Hz). The OPD could be reduced by about 70% in the frequency range between 10 and 20 Hz.

Böhn et al. [35] proposed a free-model strategy to estimate and compensate for piston aberrations due to perturbations in the optical elements by means of an accelerometer-based DFF. The vibrations displace the mirrors in the tip, tilt and piston modes, but it is the displacement in the piston mode that affects the OPD of the LBT telescope. These displacements cannot be co-regulated with the AO control loop alone, which means that the maximum resolution of the instrumentation cannot be achieved. A delay compensating algorithm introduces the necessary phase to anticipate the time delay (introduced by accelerometer signals). The method allows the use of an NGS fainter for both the fringe detector and the AO loop. Disturbances in the frequency range (8–60 Hz) were attenuated to a level less than 32% of the original level (compared to the algorithm without delay). The authors estimate that still 20% of the residual OPD caused by atmospheric aberrations and vibration in the optical elements cannot be compensated for with this type of DFF algorithms using only the main telescope mirrors, but must be done by combining feedback techniques, as well as optimizing the mechanical design, in order to reach the target of 0.1 lambda rms for the OPD.

Glück et al. [34] proposed an accelerometer-based DFF control to mitigate disturbances caused by atmospheric effects and by the telescope itself (due to fans, pumps and actuators, among others). They aimed to compensate for the shortcomings of closed-loop control systems in the observation of faint NGS with narrow bandwidths and high frequencies (>5 Hz). For this purpose, they developed an accelerometer-based DFF control and compared it with classical integral control and an LQG-based controller, respectively. The behavior of the DFF control was analyzed by means of a realistic simulation of the AO system end-to-end, taking into account real data obtained from the First Light Adaptive Optics (FLAO) of the LBT. The frequency range spanned between 0 Hz and 50 Hz, which is characteristic of the LBT. A dominant structural vibration at 13.4 Hz was observed coming from the adaptive secondary mirror. They showed that the classical integral control is not suitable for vibrations larger than 5 Hz, regardless of whether the observations are for faint or bright NGS. However, LQG control only gave good results for bright NGS and higher frequencies. The best results (SR factor increase between 2 and 4) were achieved with the DFF control in connection with a classical integral control (compared to a feedback control) over the whole frequency range.

## 4. Discussion

The main goal of this systematic review was to evaluate the evidence regarding the attenuation of telescope vibrations. To the authors’ best knowledge, this is the first systematical review investigating the methods and techniques intended for the mitigation of the effects of such vibrations. Results from the review demonstrate that the studies carried out have many characteristics in common when dealing with the problem of vibration cancellation, although when presenting the results that corroborate the benefits of the systems, there is great heterogeneity, which makes it difficult to create a pattern to classify the results in a sort of ranking.

As far as vibration suppression is concerned, in our review we have found two basic methods to deal with the problem. On one hand, we distinguish model-based estimation techniques, which assume a prior knowledge of the plant and disturbance characteristics, being sensitive to variations of such parameters [40]. For example, they assume knowledge about the structural modes and their eigenfrequencies. Classical AO systems in astronomy use an integral controller for vibration cancellation, but in many cases, it is not sufficient to achieve a high degree of suppression. In this approach, disturbance observers, such as Linear–Quadratic–Gaussian (LQG) control are often used, usually using Kalman filters, for example, to approximate the mode states. Control systems based on H∞/H2, or other DOB filters, have also been developed [41,45,46,47].

The LQG is an observer-based state feedback controller, and whose major gain obtained is due to its capability to make a good prediction [48]. According to the existing literature, the improvement of these systems with respect to the classical PI control is between 20 [49] and 30% [30]. For example, controllers based on LQG control laws have provided close-loop system performance for tip–tilt mirror control of astronomical telescopes by a factor of three or more over traditional PI control [38]. Paschall et al. [50] implemented a predictive Linear–Quadratic–Gaussian (LQG) controller and demonstrated a reduction in the rms phase distortion in the reflected wavefront from 55 to 65% within a deformable mirror AO system. Agapito et al. [39] investigated the effects of an LQG control on the LBT, while Petit et al. [51] did the same in the SPHERE instrument at the VLT. According to the results of Agapito’s study, the advantage of the LQG controller (based on Kalman filtering) is mostly for large vibration amplitudes. Le Roux [48] also used a Kalman estimator in closed-loop control for classical AO and multiconjugate adaptive optics (MCAO) to estimate the turbulence. Compared to the integrator approach, better performance is achieved in both classical AO and unseen modes estimation in MCAO. The approach works reasonably well for disturbance frequency spectra with a few very sharp and isolated peaks, since the observer tends to attenuate these peaks very well. The good results obtained by Agapito are in line with those achieved by Lozi et al. [19] on the Subaru telescope using the same type of LQG control. In another study, Lozi et al. [52] implemented an LQG (Kalman-based predictive control), using a disturbance model that was updated in real time. They demonstrated its feasibility to mitigate vibrations with changing frequencies. Similarly, Sivo et al. [53] implemented an LQG with very favorable results.

In the studies included in our review, we have seen good results when the vibration parameters are fully characterized, especially the vibration frequency peaks. Tang et al. [38] introduced a Q filter in their observer (add-on controller); Agapito et al. [39] introduced Kalman and H∞ filters, all of them having the particularity to work well when the vibration parameters were fully characterized, especially the vibration peaks. In the study of Tang et al. [38], in addition to the introduction of a conventional low-pass Q filter, they introduced two cascaded notch filters to tie the vibration peaks to the corresponding frequencies. This same idea was reproduced in the study of Niu et al. [37], whose improved EDOB achieved 36% vibration attenuation compared to the conventional PI control loop thanks to an enhanced notch filter to remove narrow-band frequencies and a modified filter to remove low frequencies.

Since the observer of this model-based approach is very sensitive to the identification of eigenfrequencies, this method cannot be used in applications where modal characteristics vary over time. This requires a major effort in implementing an online mode identification procedure [20]. To solve this, adaptive control techniques emerged, which can be easily integrated into a standard AO control architecture and can cope with variations in both plant dynamics and variations in the perturbing signal [40]. These algorithms belong to the family of adaptive vibration controllers and prior to updating the control commands; they perform an on-line estimation of the parameters (amplitude, vibration frequency and phase) and on-line estimation of the frequency response of the plant at the vibration frequency. Of all the studies included in the review, only the study by Muradore [40] implemented this technique. Previously, Di Lieto et al. [54] also implemented an adaptive vibration cancellation scheme in the fringe tracking system for stellar interferometry in large telescopes.

### 4.1. Disturbance Feedforward (DFF) and Loopback Control Techniques

Disturbance feedforward (DFF) compensation schemes usually use additional accelerometers to measure the disturbances and then use the reconstructed signal to feed the system, all within the complete control structure; that is, the actuators of the adaptive mirrors are controlled feedforward by the reconstructed signals [34]. Since the measurement of the disturbance is performed by a path independent of the control structure, the method is not limited by the sampling time delay of the image sensor [37]. This is especially useful for natural faint NGS observations, where it is necessary to increase the integration time of the WFS and therefore the bandwidth of the control loop is not sufficient to perform a good vibration cancellation. It has some disadvantages, such as the difficulty in separating useful signals even with high precision disturbance signal acquisition technology.

In Glück’s study [34], the LQG-based disturbance observer (with a Kalman filter) did not achieve the best results for the case of faint NGS. Therefore, they had to employ an additional accelerometer-based DFF control (in connection with classical integral control) to increase the SR by a factor up to 4. This same scheme was used by Böhm [20] for the estimation and correction of the OPD in the LBTI. Kalman estimation had not worked correctly for the authors in a previous study [55] due to the characteristic spectrum of the LBT since the modes were too close together for the estimator to distinguish them.

Thus, the results obtained both in the included studies and in the literature suggest the use of feedforward techniques in combination with classical feedback control techniques [35,56] to improve the performance of the vibration suppression algorithms in telescopes.

### 4.2. AO Systems and Atmospheric Turbulence

AO has proven to be a fairly robust technique to compensate for the effect that atmospheric turbulence has on the images generated at the telescopes [57,58,59,60,61]. Some studies included in this review, in addition to considering all structural vibrations due to phenomena such as wind, etc., have introduced the effect that atmospheric turbulence introduces in wavefronts [39,40]. The different atmospheric turbulence profiles and their associated parameters are widely used in the development of AO systems. In fact, the image quality is highly dependent on the turbulence velocity, often determined by the wind speed at a pressure level of 200 hPa (V200). The suitability of a given site for good astronomical observations is often evaluated using the information provided by V200, and this information is useful for making recommendations for AO systems [62]. Some authors have made astroclimatic characteristics for different observatory sites in order to be used to plan the observing time, as well as to facilitate the development of AO systems [63,64], since, for example, the vertical wind speed profiles are one of the most important characteristics for the determination of the dynamic range of an adaptive optics system [62]. For example, Goodwin et al. [65] developed a model of the optical turbulence profile (model-OPT) to be used as input to simulations to evaluate the performance of the AO system of the Giant Magellan Telescope [5].

In summary, although the Kolmogorov model has been and is being widely used as a model of atmospheric turbulence, there are authors who have found deviations with respect to the real atmospheric spectrum, especially as the aperture of the telescopes increases [66,67]. According to Martínez et al. [68], it is necessary to work on other atmospheric models for modern telescopes with interferometers with hundreds of meters, such as the Von Kárman model [69], although there is no evidence yet that it is sufficiently accurate. 

### 4.3. Simulated vs. Real Telescope Data

In the studies considered in this review, we have found that all of them use experimental laboratory setups. Although some of them have taken real data from certain telescopes, they mainly present their results by means of simulations. For these reasons, it has not been taken into account that one of the major difficulties in developing optimal systems for vibration control in telescopes is the vibrations occurring at the time of transit, whose nature is unpredictable [19]. This point is critical because most of the field rotation necessary for post-processing algorithms is provided by this transit time. According to Lozi et al. [19], this vibration frequency, besides having a high amplitude, is difficult to simulate and is accompanied by a high amplitude and short and random transient events, which makes its correction even more difficult.

### 4.4. Limitations

This review has limitations that are worth mentioning. The final number of studies meeting the inclusion criteria was modest. As far as the outcome measures were concerned, there was no uniformity among the studies in the parameters to measure the effect of vibration attenuation of the different techniques. Therefore, a greater number of studies would be needed, as well as greater homogeneity in the results to corroborate whether the techniques applied are really effective (and to what extent) in the rejection of vibrations in astronomical infrastructures.

In addition, all the studies analyzed have been carried out in controlled laboratory environments, in which the AO systems have been reproduced, and although some of them have used real data, it has not been possible to test the effectiveness of the solutions at the observatory facilities (in situ tests). We are aware of the technical difficulty of testing in astronomical facilities, but it would be interesting to test the systems in these real environments. For example, in many cases it has not been possible to verify the incidence of the atmosphere on the aberration of the incident beam. AO systems need this information about the distortion that the atmosphere introduces on the wavefronts. Only a few studies [34,39] have simulated the behavior of the atmosphere through a turbulence model in order to validate the proposed algorithms.

### 4.5. Implications for Research

Our review suggests that there are still several important implications for further research in the field of telescope vibration attenuation. Therefore, some issues are demanding future research, such as the following:

- As discussed above, the results of further research into vibration attenuation should be aimed at yielding results in widely used parameters, such as SR, a measure of the quality of a telescope in the field of astronomy.
- We consider it extremely important to take into consideration the atmospheric data of the different sites in order to improve the adaptive optics systems by using the different atmospheric turbulence profiles. In this way, the systems would be calibrated according to the sky in which they operate [70,71,72,73,74,75,76]. Control applications also should be developed taking into account the particularities of each telescope and environment where it is currently operating. It is interesting to explore new atmospheric turbulence models other than the Kolmogorov model, and to do so for large telescopes and stellar optical interferometers, such as the one proposed by Jia et al. [77]. Therefore, we propose research on more robust systems that include all possible sources of beam aberration due to atmospheric perturbations, natural phenomena and structural vibrations, and especially the tuning of these systems according to the conditions of each telescope.
- On the other hand, an interesting topic that has been addressed only in one study deals with the need for a longer exposure time (exposure time in the main image camera and wavefront sensor) when observing faint stars, in order to achieve high-contrast imaging. This leads to a minimum sampling time in the AO compensation setup in digital control systems (Nyquist frequency), and thus reduces the maximum vibration frequency that the system can attenuate [78]. This is especially critical with faint NGS. Thus, there is a trade-off between maximum frequency to attenuate and exposure time. More work with different NGS would be necessary to determine frequency ranges with an acceptable compromise in vibration attenuation. It would also be interesting to characterize the quality of the AO systems taking into account the magnitude of the stars and the seeing conditions, respectively.
- Most of the studies focus on the first vibration modes (tip and tilt, fundamentally) because they are the ones that contribute the most to the wavefront deformation. Only the study of Muradore et al. [40] also adds the study with the focus, trefoil-x and trefoil-y modes. Therefore, it may be necessary to investigate further the consequences of higher modes of vibration (simulated through the moments via Zernique polynomials, for example). In the case of an optical interferometer, an important component in the contribution of atmospheric turbulence, is given by the higher-order Zernike modes [79] (wavefront corrugations over each individual telescope aperture), in addition to the differential piston-mode Zernike component [80].
- In the analyzed studies, little or nothing has been said about the quality of the images obtained in the telescopes and the real influence of the vibrational effect. It is important to quantify precisely the effect on the image, taking into account the maximum permissible resolutions in each of the different telescopes. The idea is to reach a compromise between increasing the complexity of the vibration cancellation system and the final quality of the image (cost-effectiveness), because the final product provided by astronomical observatories is the image.
- As pointed out by Lozi et al. [19], some other technique should be explored, for example, those that merge multiple wavefront sensors measurements and accelerometers, such as sensor fusion, to improve mitigation of vibrations. Like-wise, predictive control algorithms are called for to offer improvement alternatives [81,82], since this type of algorithm can predict the vibrations for the telescope pointing, using this information to offer the best correction.

## 5. Conclusions

To the best of our knowledge, this is the first systematic review investigating the different methods to cope with the effects of vibration on telescopes. The results obtained confirm the good performance of AO control systems, although it is true that most of the results come from simulations obtained with laboratory experimental setups. We highlight that more studies are needed to verify the benefits of AO systems, and that these would need to be tested in different astronomical observatory locations, since it is necessary to transfer the good results obtained in experimental laboratories to real telescopic structures (in situ tests). Knowledge of atmospheric turbulence is essential for optimal performance of AO systems. In addition, it is necessary to unify the results through more homogeneous measurements that give us a better idea of the degree of improvement of each of the proposed systems.

## Figures and Tables

**Table 1:** Characteristics of the included studies.

Author(s)/ Year of Publication	Type of Telescope	Effect to be Compensated	Sensing Technology/ Sensor Location	Goal of Study	Techniques and Methods/Frequencies	Outcome Measures	Conclusions
Agapito et al., 2011 [39]	LBT	Atmospheric turbulences/telescope structure vibrations	Acquisition camera (CCD47)	to suppress disturbances in tip–tilt modes	Experimental Laboratory to simulate the LBT-AO and experimental test using a Solar tower Comparison among classical integrator controller and observer-based techniques based on Kalman and H∞ filters Frequency range: 0 Hz–20 Hz	Under turbulence and vibration conditions (both simulated) the integral controller deteriorates SR at 2.2 um up to 30.9 % whereas both Halman and H∞ provided a SR about 80% The mixed-Kalman controller has a SR about 70% at 2.2 um for amplitudes from 0 to 150 mas (experimentally tested)	Kalman and H∞ mixed filter perform better than classic integrator controller (especially Kalman) in terms of SR Both are more robust against frequency errors To obtain the best results vibration must be precisely characterized ( frequency value)
Böhm et al., 2014 [20]	LBT	Wind/Telescope structure	Three accelerometers in optical axis direction and two accelerometers in mirror plane/mirror surface	To minimize the effects of vibrations on the telescope mirrors by reducing the optical pathway difference (OPD)	Simulation laboratory to imitate the vibration behavior at the LBT Accelerometer DFF compensation scheme Two different approaches in the control loop: one to reconstruct the position of the mirror from the accelerometer measurement (model-based reconstruction) and another using a broad-band filter Frequency range: 8 Hz–80 Hz	The OPD was reduced about 80% in the range 14–20 Hz	Model-based reconstruction works better with single frequency vibrations (periodic excitation) The filter approach is more flexible and works better in the 8 to 80 Hz range
Muradore et al., 2014 [40]	ESO-VLT	Atmospheric turbulences/telescope structural vibrations	WFS	Vibration rejection on tip–tilt modes in AO system	Experimental Laboratory/Real working conditions at the telescope Adaptative Vibration Cancellation (AVC) in AO system Frequency range: 0 Hz–200 Hz	Rejection larger than 20 dBs (on average) at vibration peaks of 18 Hz and 48 Hz (in PSD function) Tip–tilt residual was reduced from 15.2 mas to 11 mas (rms) In the GALACSI simulated AO system, the LE Strehl with the AVC improved the relative Strehl from 0.64 to 0.96. Residual jitter was reduced from 7.1 to 2.2 (mas rms)	The vibration cancellation algorithm reduced residual vibrations to the noise level (in the analyzed scenarios) The modular implementation allows the rejection of multiple vibration peaks in multiple modes running in parallel with the current AO control loop The algorithm is independent of the knowledge of the dynamics of the AO system and no operator supervision is required
Glück et al., 2017 [34]	LBT	Structural vibration	Accelerometer/Mirror	To compensate vibrations (by improving the AO performance for faint NGS)	Experimental Laboratory with real data from FLAO Comparison of conventional integral control, LQG, and an accelerometer-based DFF control Frequency range: 0 Hz–50 Hz	SR was increased by a factor 2 to 4 with integral and DFF control (in comparison with the feedback control) EE constant over the frequency range for DFF control for both bright and faint NGS (0–50 Hz)	Conventional integral control is sufficient for bright NGS and frequencies above 10 Hz, but not useful for faint NGS and frequencies above 5Hz The LQG performed well with bright NGS and frequencies above 5Hz. DFF controller together with integral controller obtained the best results by compensating vibrations over the entire frequency range
Böhm et al., 2017 [35]	LBT	Structural vibration on optical components	Five accelerometers (three in Z-axis and two for piston and tip–tilt modes respectively)/mirror surface	To estimate and compensate piston aberrations due to vibrations of optical components	Accelerometer DFF with delay compensation Frequency range: 8 Hz–60 Hz	Disturbances in the frequency range attenuated to a level less than 32% of the original level (compared to the algorithm without delay)	A delay compensating algorithm introduce the necessary phase to anticipate the time delay (introduced by accelerometer signals) To further decrease the remaining OPD feed forward and feedback techniques should be applied There is still a residual OPD due to internal instrument vibration and atmospheric aberrations Delay compensation necessary even for small delays
Tang et al., 2018 [32]	Optical telescopes	Structural vibrations	Image sensor	to reduce structural vibrations in tip–tilt mirror modes	Laboratory experimental setup Closed-loop control based on Youla–Kucera parametrization with Q_31_ filter and two notch filters Frequency range: 0 Hz–78 Hz	Low frequency (0-10 Hz) vibration attenuation 33.8% less with respect to the integral controller Closed-loop errors 36% less compared to the classical integrator when adding tow notch filters at 22 Hz and 48 Hz, respectively	A low pass filter (Q31-filter) and a band-pass filter (two notch filters) necessary to attenuate vibration along the frequency range The scanning method ensures the detection of the vibrations of interest, which are necessary for the notch filters design The closed-loop bandwidth is not widened but error attenuation is enhanced
Tang et al., 2019 [36]	Optical telescopes	Telescope structure	Image sensor	To reject structural vibrations in tip–tilt mirror modes	Laboratory experimental setup A DOB control with an improved Q-filter added into the original loop for tip–tilt mirror control system Frequency range: 0 Hz–21 Hz	Vibration attenuation (with the DOB) less than 46.1% with respect to the integral controller The DOB with an improved band pass filter to mitigate multiple narrowband vibration attenuated about 1/3 compared to the conventional integral control	This improved DOB is not constrained by a precise model so that noise in the loop cannot seriously influence vibration mitigation The closed-loop bandwidth is not widened but the disturbance attenuation is improved
Niu et al., 2019 [37]	Optical telescope	Structural vibrations	Image sensor	To mitigate wideband vibrations of tip–tilt mirror	Experimental Laboratory Improved EDOB based on Youla parametrization in the tip–tilt mirror system, incorporating a Q-filter with 3 notch filters Frequency range: 7.5 Hz–12.5 Hz	The improved EDOB with three notch filters achieved 36% vibration attenuation compared to conventional PI control loop	The system only uses an image sensor for position deviation (cost-effective) Due to a low dependence on the system model, the vibration rejection ability is not restricted by the noise in the loop Due to its simplicity in design and structure, the system could be used in other servo control systems
Tang et al., 2002 [38]	Optical telescope	Telescope structure	Image sensor	To mitigate vibrations in tip–tilt mirror	Laboratory experimental Add-on controller built with only the position error Improved Q-filter combining low-pass filter and notch filter Frequency range: 0 Hz–11 Hz	The PI controller with add-on controller and Q-filter (low pass filter) can mitigate up to10% of the original error compared to PI controller The PI controller with add-on controller and Q-filter with low pass filter and three cascaded notch filters can mitigate up to 43% of the original error (compared to PI controller)	The results obtained allow a significant reduction of the perturbations in the mirror tip–tilt compared to a PI controller The design of notch filters requires prior knowledge of the center frequencies of vibrations with higher energy bands No increase in closed-loop bandwidth but improvement in disturbance attenuation

AO: Adaptive Optics; AVC: Adaptive Vibration Canceller; CCD: Charge-Coupled Device; DFF: Disturbance Feedforward; DOB: Disturbance Observer; EDOB: Error-based Disturbance Observer; E-ELT: European-Extremely Large Telescope; ESO: European Southern Observatory; FLAO: Fist Light Adaptative Optics; LBT: Large Binocular Telescope; LE Strehl: Long Exposure Strehl; LGS: Laser Guide Star; LQG: Linear-Quadratic-Gaussian control; mas: milli-arcseconds; NGS: Natural Guide Star; OPD: Optical Pathway Difference; PI: Proportional-Integral; PSD: Power Spectral Density; SR: Strehl Ratio; VLT: Very Large Telescope; WFS: Wavefront Sensor.

## Data Availability

Not applicable.
